# *PCLO* Is Associated with Tumor Mutational Burden and Immunity in Patients with Oral Squamous Cell Carcinoma

**DOI:** 10.3390/cimb47060426

**Published:** 2025-06-06

**Authors:** Yuping Liu, Ye Zhang, Lijing Zhu, Zheng Zhou, Yajuan Cui, Heyu Zhang, Chuanxiang Zhou

**Affiliations:** 1 Department of Oral Pathology, Peking University School and Hospital of Stomatology, Beijing 100081, China; liuyuping@stu.pku.edu.cn (Y.L.); zlj@stu.pku.edu.cn (L.Z.); jackloveslife@126.com (Z.Z.); cuiyajuan998@163.com (Y.C.); 2 Department of Stomatology, Beijing Chaoyang Hospital, Capital Medical University, Beijing 100020, China; zuiaizaizhonghero@126.com; 3 Central Laboratory, Peking University School and Hospital of Stomatology, Beijing 100081, China; zhangheyu1983@sina.com

**Keywords:** oral squamous cell carcinoma, *PCLO*, tumor mutation burden, immune cell infiltration, piccolo

## Abstract

To determine predictive biomarkers for prognosis by analyzing the association between tumor mutational burden (TMB) and mutant genes in patients with oral squamous cell carcinoma (OSCC) and to validate *PCLO* as an OSCC predictive biomarker, OSCC genetic mutation data were downloaded from The Cancer Genome Atlas (TCGA) and the International Cancer Genome Consortium (ICGC) database. Immune cell infiltration analysis and visualization were performed using R software. The relationships between overall survival (OS) and mutant genes or clinicopathological factors were investigated by Kaplan–Meier analysis and Cox regression analysis, respectively. Gene set enrichment analysis (GSEA) was used to explore the associations between mutant genes and functional pathways. Immunohistochemistry was performed to verify the presence of the piccolo protein in OSCC tissues. Finally, 17 mutated genes shared between TCGA and the ICGC database were detected. The TMB in the *PCLO*-mutated group was found to be significantly greater than that in the *PCLO* wild-type group, and *PCLO* mutation was associated with poor OS. Cox regression analysis revealed that *PCLO* is a significant prognostic factor for OSCC. GSEA and immune cell infiltration analysis revealed that *PCLO* is associated with the immune system, which suggests that *PCLO* mutation might affect the immune response. *PCLO* expression was considerably higher in OSCC tissues with *PCLO* mutations than in corresponding normal epithelium tissues and OSCC tissues without *PCLO* mutations (*p* < 0.05). *PCLO* mutation could serve as a promising predictive biomarker for prognosis in patients with OSCC.

## 1. Introduction

Oral squamous cell carcinoma (OSCC) is a malignant neoplasm arising from the mucosal epithelium of the oral cavity and exhibits variable squamous differentiation [1]. It is the 16th most common malignant tumor worldwide [2]. According to the World Health Organization International Classification of Diseases (ICD-11) [1], OSCC includes squamous cell carcinoma arising from any oral mucosal site and squamous cell carcinoma of other or ill-defined sites in the lip, oral cavity, or pharynx. In 2020, ~380,000 new cases of OSCC were diagnosed globally [3]. In high-risk countries such as Sri Lanka and India, OSCC may account for up to 25% of all new cancer cases [4]. The most important etiological factors in OSCC are tobacco use, excess alcohol consumption, and betel quid usage [4]. There are a number of traditional treatment methods for patients with OSCC, including surgery, radiotherapy, and chemotherapy. However, the treatment efficacy of these traditional methods is limited [5]. The emergence of targeted molecular therapies has reduced the side effects of nonspecific cell death and improved survival rates in patients with OSCC [6]. In recent years, researchers in various fields have focused more on the molecular mechanism of tumors to identify effective biomarkers. Numerous biomarkers, such as FAT atypical cadherin 1 (*FAT1*) [7] and circulating long non-coding RNAs [8], have been identified, and have been suggested to have good potential or diagnostic performance in OSCC treatment.

*PCLO* encodes piccolo protein, which is part of the presynaptic cytoskeletal matrix, is involved in establishing active synaptic zones and in synaptic vesicle trafficking. Previous studies have shown *PCLO* is mainly associated with depressive disorder [9]. In recent years, *PCLO* has been discovered to be frequently mutated in cancers, such as esophageal squamous cell carcinoma (ESCC), gastric cancer and hepatocellular carcinoma [10,11,12].

The tumor mutational burden (TMB), measured as the number of mutations that exist within a megabase of genomic territory, is an emerging and commonly used biomarker for the prediction of immune system therapy response [13]. It has been suggested that a greater frequency of gene mutations in cancers leads to a high TMB, increasing the likelihood of generating immunogenic tumor neoantigens recognized by the host immune system [14,15]. Some evidence suggests that the TMB can serve as a therapeutic biomarker in patients with lung cancer, head and neck cancer, or bladder cancer [16,17,18].

The present study aimed to explore potential biomarkers for clinical prognosis by analyzing the association between TMB and mutant genes in patients with OSCC, and to determine whether *PCLO* is an OSCC biomarker. Somatic mutation, transcriptome, and clinical data of patients with OSCC were collected from The Cancer Genome Atlas (TCGA) and somatic mutation data were collected from the International Cancer Genome Consortium (ICGC) database. After screening the common high-frequency mutated genes in the two databases, the present study further explored the relationships between these gene mutations and TMB, as well as prognosis. Immunohistochemistry was performed for further exploration.

## 2. Materials and Methods

### 2.1. Data Collection

Somatic mutation and transcriptome data for samples from patients with OSCC (n = 372) and Indian patients with OSCC (n = 171) were acquired from TCGA (https://portal.gdc.cancer.gov/ (accessed on 12 September 2022); study abbreviation, HNSC) and the ICGC (https://dcc.icgc.org/ (accessed on 12 September 2022)) database [dataset (ORCA-IN) ORAL CANCER-IN.], respectively. The clinical data associated with 372 OSCC samples were obtained from TCGA. No clinical data were available in the ICGC database. The data were extracted and sorted using Perl software (v5.30.0.1; https://www.perl.org/ (accessed on 11 September 2022)), and analyzed using R (v4.1.3; https://www.r-project.org/ (accessed on 11 September 2022)). Regarding the clinical data, only patients with OSCC whose complete information was available were included, and patients whose survival status, age, sex, grade, or TNM stage data were missing were excluded. To visualize the characteristics of mutations in the two databases, waterfall plots were produced using the R package ‘GenVisR’ (release no. 3.19; https://www.bioconductor.org/packages/release/bioc/html/GenVisR.html (accessed on 12 September 2022)). Using the R package ‘Venn’ (v0.1.10; https://cran.r-project.org/web/packages/ggvenn/index.html (accessed on 14 September 2022)), the mutated genes shared between the two databases were obtained. Sequencing data used to identify patients with *PCLO* mutations can be found in the Genome Sequence Archive at the BIG Data Centre of the Beijing Institute for Genome Research, Chinese Academy of Sciences, under accession number PRJCA002133 (https://ngdc.cncb.ac.cn/gsa-human/browse/HRA000107 (accessed on 8 February 2023)).

### 2.2. Calculation of TMB in TCGA Patients with OSCC

TMB was defined as the number of insertions, deletions (indels), replacement mutations, and gene coding errors per megabase in the evaluated coding regions of the genome [19]. Patients with synonymous mutations were removed. To calculate the TMB, the absolute number of somatic mutations was divided by the exome size. Subsequently, the relationship between the TMB and shared mutated genes was obtained via assessment and visualization using the R package ‘ggpub’ (v0.6.0; https://cran.r-project.org/web/packages/ggpubr/index.html (accessed on 14 September 2022)).

### 2.3. Gene Set Enrichment Analysis (GSEA) in Patients with OSCC from TCGA

GSEA software 4.3.1 (https://www.gsea-msigdb.org/gsea/index.jsp (accessed on 20 September 2022)) was used to identify significantly different functional pathways according to the *PCLO* mutation level, including the mutated group and the wild-type group. A nominal *p*-value < 0.05 or a normalized false discovery rate of <0.25 was regarded as the cutoff criterion.

### 2.4. Immune Cell Infiltration in Patients with OSCC from TCGA

The ‘CIBERSORT’ package (v1.03) of R software (https://cibersortx.stanford.edu/ (accessed on 21 September 2022)) was used to calculate the fractions of each immune cell in each OSCC sample with gene expression data, and *p* < 0.05 was regarded as the cutoff criterion [20]. A correlation heatmap was used to analyze the correlation of each type of immune cell with the other types in OSCC samples using the ‘corrplot’ package (v0.92; https://cran.r-project.org/web/packages/corrplot/index.html (accessed on 21 September 2022)). The R package ‘vioplot’ (v0.5.0; https://cran.r-project.org/web/packages/vioplot/index.html (accessed on 21 September 2022)) was used to visualize the differences in the infiltration of 22 immune cell types between *PCLO*-mutated and *PCLO*-wild-type samples. *p* < 0.05 was employed as the standard to identify the immune cell populations possibly influenced by the mutation levels of *PCLO*.

### 2.5. Immunohistochemistry (IHC) of 5 Patients with OSCC with PCLO Mutations

The exome sequencing data were obtained in our previous study [21]. Patients with OSCC with or without *PCLO* mutation were identified using the sequencing data. The OSCC tissues were divided into four groups: OSCC without *PCLO* mutation and its normal epithelium, and OSCC with *PCLO* mutation and its normal epithelium. The present study was approved by the Institutional Review Board of the Peking University Hospital of Stomatology (approval no. PKUSSIRB-2024104193; Beijing, China). The tissues were fixed with 4% paraformaldehyde overnight at room temperature. Paraffin-embedded tumor tissues were collected from 10 patients with OSCC (date range of recruitment, August 2009–November 2014; 8 male and 2 female patients; mean age, 52.3 years; age range, 30–62 years) who were diagnosed at the Oral Pathology Department of the Peking University Hospital of Stomatology (Beijing, China). The inclusion criteria for the OSCC with *PCLO* mutation group were: patients with *PCLO* mutations from the data from Zhang et al. [21]. The normal epithelium group inclusion criteria were: normal mucosa from patients with *PCLO* mutations. The inclusion criteria for the OSCC without *PCLO* mutation group were: patients without *PCLO* mutations and with relatively sufficient tumor tissue remaining for the production of good quality sections. The exclusion criteria were: insufficient tissue remaining to complete the experiment. Tissue sections (3 μm-thick) were deparaffinized with xylene and rehydrated in descending graded ethanol. The sections were subsequently submerged in sodium citrate buffer (pH = 6) and the antigens were repaired at a high temperature (95–100 °C) and high pressure for 2.5 min. The sections were then treated with endogenous peroxidase blockers (undiluted; PV-9005; OriGene Technologies, Inc., Wuxi, China) to quench the endogenous peroxidase activity for 10 min. To block nonspecific binding, the sections were incubated with 10% goat serum albumin (Beijing Solarbio Science & Technology Co., Ltd., Beijing, China) for 30 min at room temperature. The sections were incubated with rabbit anti-piccolo (1:200; ab20664; Abcam, Cambridge, UK) at 4 °C overnight. Finally, the sections were treated with goat anti-mouse/rabbit IgG HRP-polymer (undiluted; GK600711A; Gene Tech Co., Ltd., Shanghai, China) for 20 min at room temperature. 3,3′ Diaminobenzidine was used as the chromogen after washing with distilled water for 3 min at room temperature. The sections were then counterstained with hematoxylin for 10 s at room temperature. Tissue staining was observed under a light microscope. Image-Pro Plus 7.0 software (Media Cybernetics, Inc., Rockville, MD, USA) was used to select areas with equal brown and yellow staining as a uniform standard for assessing positive results in all images. IHC results for *PCLO* were evaluated using the average optical density (AOD) [22].

### 2.6. H&E Staining

Tissues were fixed with 4% paraformaldehyde overnight at room temperature, and paraffin-embedded tumor tissues were collected from 10 patients with OSCC. Tissue sections (4 μm-thick) were deparaffinized with xylene and rehydrated in descending graded ethanol. Sections were immersed in hematoxylin differentiation solution (undiluted; G1039-500ML; Wuhan Servicebio Technology Co., Ltd., Wuhan, China) for 10 s at room temperature, and then sections were immersed in hematoxylin reblue solution (undiluted; G1040-500ML; Wuhan Servicebio Technology Co., Ltd.) for 10 s at room temperature, then rehydrated in ascending graded ethanol and xylene. Tissue staining was observed under a light microscope.

### 2.7. Statistical Analysis

The OSCC data were extracted and organized in Perl (v5.30.0.1; https://www.perl.org/ (accessed on 11 September 2022)), so that they could be analyzed in R software (v4.1.3; https://www.r-project.org/ (accessed on 11 September 2022)) to complete all the statistical analyses. Survival analyses were performed using TCGA data. The relationships between overall survival (OS) and 13 common frequently mutated genes were assessed and visualized using Kaplan–Meier curves, and the associations were analyzed by log-rank tests. The genes with no statistically significant association with OS were filtered out. Univariate and multivariate Cox regression analyses were used to investigate the associations between clinicopathologic characteristics (age, sex, grade, stage, TMB, and *PCLO* status) and OS. In multivariate Cox regression models, we determined whether *PCLO* could be considered an independent predictor based on whether the confidence interval (CI) for the hazard ratio (HR) of *PCLO* did not contain 1 and, in conjunction with this, the corresponding *p*-value reached a level of significance (usually *p* < 0.05). The Wilcoxon rank-sum test was used to investigate the differences in immune cells between the *PCLO* wild-type and *PCLO*-mutant groups. Pearson correlation analysis was used to investigate the correlations between different immune cell types. Comparisons of AOD values between the without *PCLO* mutation group and the *PCLO* mutation group were performed using two-way mixed ANOVA with Bonferroni post hoc test. The data are reported as the mean ± standard error of the mean. *p* < 0.05 was considered to indicate a statistically significant difference.

## 3. Results

### 3.1. Overview of Mutated Genes in OSCC

**Figure 1:**
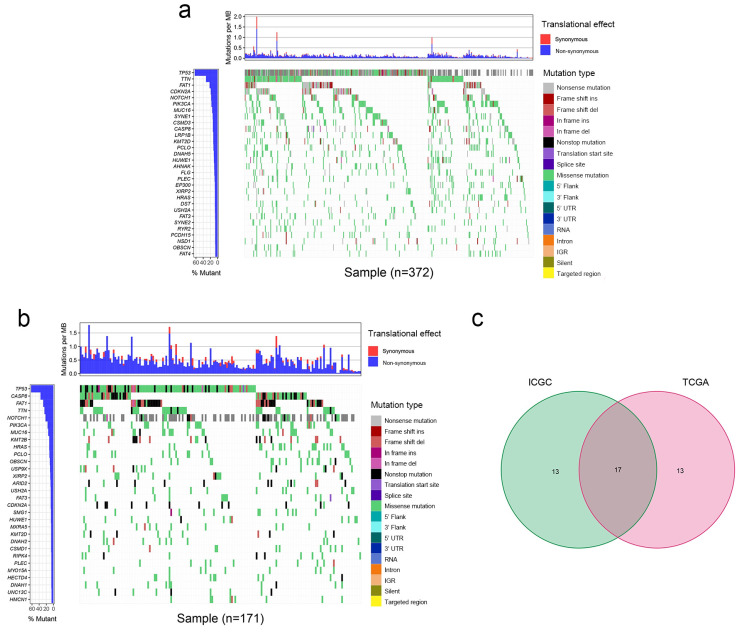
Mutation profile landscape in patients with OSCC. (**a**) Frequently mutated genes in OSCC specimens in TCGA are shown in the waterfall plot. Genes ranked by mutation frequency are presented in the left panel. The right panel shows the variety of mutation types. (**b**) Frequently mutated genes in the OSCC samples from the ICGC dataset are depicted as a waterfall plot. The left panel shows the mutation frequency and corresponding genes. The right panel shows the multiple mutation types of these genes. (**c**) Common frequently mutated genes in the ICGC and TCGA cohorts are shown in the Venn diagram. Del, deletion; ICGC, International Cancer Genome Consortium; IGR, intergenic region; Ins, insertion; MB, megabase; OSCC, oral squamous cell carcinoma; TCGA, The Cancer Genome Atlas; UTR, untranslated region.

The mutation data of patients with OSCC were downloaded from TCGA and the ICGC database. The top 30 frequently mutated genes with their corresponding mutation profiles in TCGA and the ICGC database were identified (Figure 1a,b). In addition, 17 frequently mutated genes were identified by generating a Venn diagram indicating the intersection of the top 30 most frequently mutated genes in the ICGC and TCGA cohorts, and these were *TP53*, *CASP8*, *FAT1*, *TTN*, *NOTCH1*, *PIK3CA*, *MUC16*, *HRAS*, *PCLO*, *OBSCN*, *XIRP2*, *FAT3*, *CDKN2A*, *USH2A*, *HUWE1*, *KMT2D*, and *PLEC* (Figure 1c). In the subsequent analyses, these 17 commonly mutated genes were the focus of the present study.

### 3.2. PCLO Mutation Status Is Associated with TMB and OS

**Figure 2:**
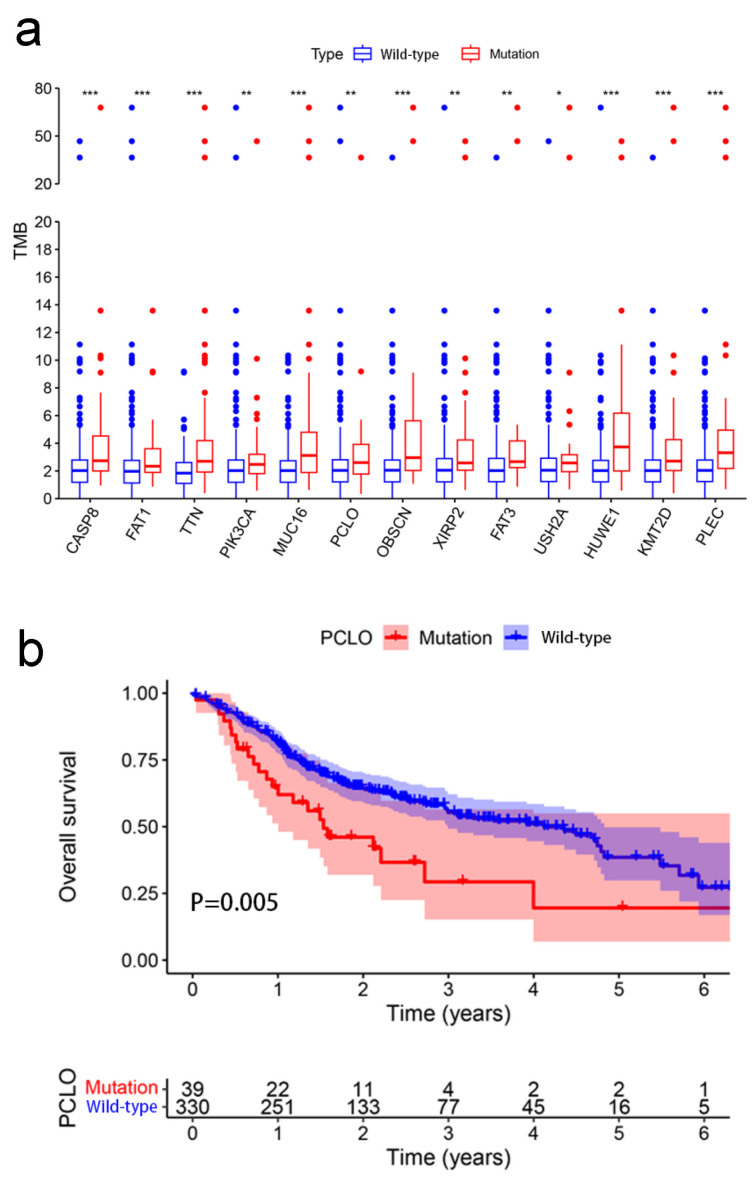
Association between TMB and mutant genes. (**a**) Mutations of 13 genes were associated with TMB. The Wilcoxon rank-sum test was used. * *p* < 0.05; ** *p* < 0.01; *** *p* < 0.001. (**b**) Association between gene mutations of *PCLO* and overall survival. Kaplan–Meier survival analysis of patients with oral squamous cell carcinoma revealed relationships between *PCLO* mutations and prognostic outcomes. The *p*-value is indicated in the plot. TMB, tumor mutational burden.

**Figure 3:**
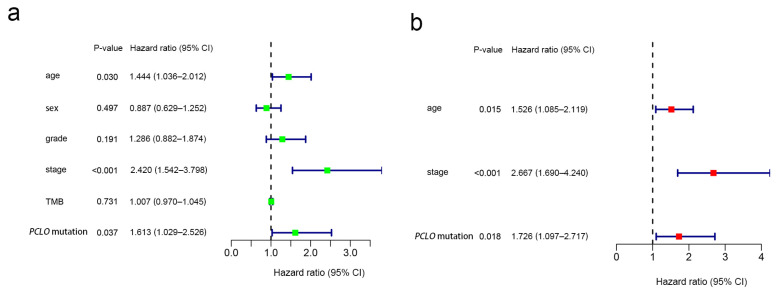
Cox regression analysis. (**a**) Univariate and (**b**) multivariate overall survival analyses of patients with oral squamous cell carcinoma revealed that *PCLO* mutation was significantly associated with survival status. TMB, tumor mutational burden.

Patients with OSCC were classified into two groups according to the mutation status of the examined genes (wild-type and mutant group). Among the 17 mutated genes analyzed, four were not significantly related to the TMB and were therefore removed. Samples with mutations in the remaining 13 genes (*CASP8*, *FAT1*, *TTN*, *PIK3CA*, *MUC16*, *PCLO*, *OBSCN*, *XIRP2*, *FAT3*, *USH2A*, *HUWE1*, *KMT2D*, and *PLEC*) exhibited a significantly greater TMB than those in the wild-type group (Figure 2a). A higher TMB frequently indicates a favorable clinical prognosis for patients with OSCC [23]. Consequently, it was assessed whether these gene mutations associated with higher TMB also influenced the survival outcome of patients with OSCC by performing Kaplan–Meier analysis. *FAT1* (*p* = 0.003), *PCLO* (*p* = 0.005), and *KMT2D* (*p* = 0.019) mutation was associated with OS, whereas the mutations in the other 10 genes appeared not to be significantly associated with OS (*p* > 0.05) (Figure S1). *PCLO* is relatively understudied in OSCC, and thus, it was explored further. As shown in Figure 2b, patients with OSCC with *PCLO* mutations had a worse OS than noncarriers. Thus, *PCLO* could be a novel biomarker for evaluating the survival prognosis of patients with OSCC. The results of the univariate regression analysis indicated that several clinicopathological factors, including age [hazard ratio (HR), 1.444; 95% CI, 1.036–2.012; *p* = 0.030], stage (HR, 2.420; 95% CI, 1.542–3.798; *p* < 0.001), and *PCLO* mutation status (HR, 1.613; 95% CI, 1.029–2.526; *p* = 0.037), were significantly associated with OS (Figure 3a). As shown in Figure 3b, *PCLO* mutation remained a significant prognostic factor after taking age and stage into account; thus, *PCLO* mutation was regarded to be an independent prognostic factor for OS in patients with OSCC. Age and stage were also significant prognostic factors.

### 3.3. GSEA for PCLO Mutation

**Figure 4:**
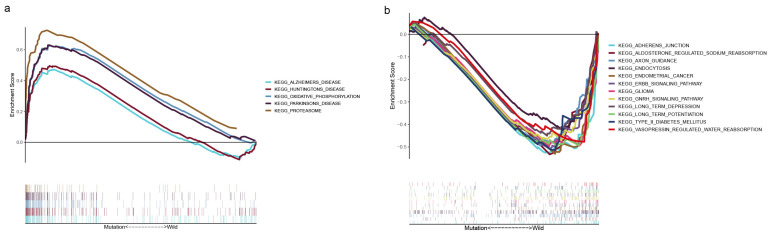
Gene set enrichment analysis. (**a**) Multiple gene enrichment plot showing that a series of gene sets were enriched in the *PCLO*-mutant group. (**b**) Multiple gene enrichment plot showing that a series of gene sets were enriched in the *PCLO* wild-type group. KEGG, Kyoto Encyclopedia of Genes and Genomes.

The results of GSEA based on the TMB data showed that the *PCLO* mutation may be associated with the activation of ‘Alzheimers disease’, ‘Huntingtons disease’, ‘oxidative phosphorylation’, ‘Parkinsons disease’, and the ‘proteasome’ pathway (Figure 4a). GSEA results were also obtained for the *PCLO* wild-type group, as shown in Figure 4b; pathways such as ‘adherens junction’, ‘aldosterone regulated sodium reabsorption’, ‘axon guidance’, ‘endocytosis’, ‘endometrial cancer’, ‘ERBB signaling pathway’, ‘glioma’, ‘GNRH signaling pathway’, ‘long term depression’, ‘long term potentiation’, ‘type II diabetes mellitus’, and ‘vasopressin regulated water reabsorption’ were significantly enriched.

### 3.4. Relationship Between Immune Cell Infiltration and PCLO Mutation Status

**Figure 5:**
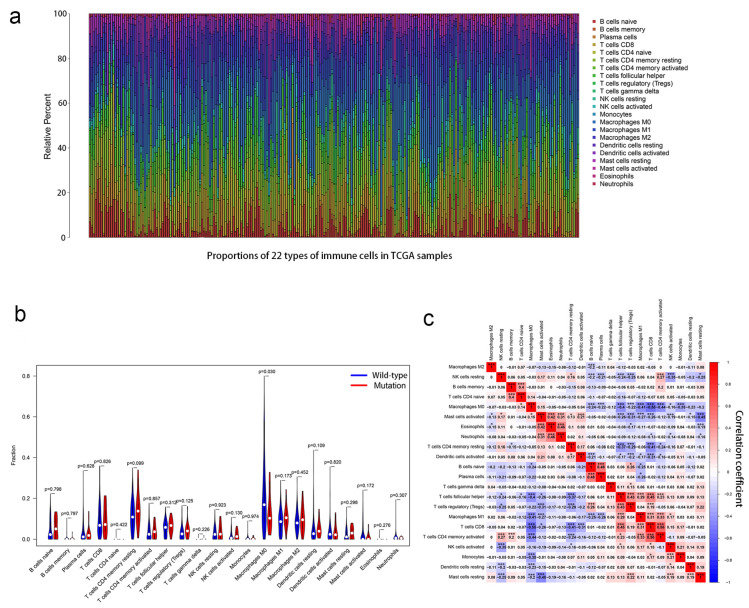
Association between *PCLO* mutations and the proportions of 22 types of immune cells. (**a**) Stacked bar chart showing the proportions of 22 types of immune cells for every sample. (**b**) Violin plot showing the different proportions of the 22 immune cell types in *PCLO*-mutant and *PCLO* wild-type oral squamous cell carcinoma samples. Blue indicates the *PCLO* wild-type group, and red indicates the *PCLO*-mutant group. The Wilcoxon rank-sum test was used. (**c**) Correlations between different immune cell types showing the correlations of 22 immune cell types in OSCC samples from the dataset from TCGA. Red denotes a positive correlation, while blue denotes a negative correlation. Pearson correlation analysis was used. * *p* < 0.05; *** *p* < 0.001. NK, natural killer; TCGA, The Cancer Genome Atlas.

Based on GSEA enrichment analysis, we found that the *PCLO* mutant group was enriched for immune-related pathways. Therefore, the immune cells of OSCC patients were further analyzed. Using the CIBERSORT algorithm, the proportions of 22 types of immune cells in the tumor microenvironment of patients with OSCC were calculated (Figure 5a). The violin plot showed that the number of M0 macrophages in patients was significantly lower in the *PCLO* mutation group than in the wild-type group (Figure 5b). The immunocyte correlation matrix showed that M0 macrophages had the strongest negative correlation with CD8 T cells (r = −0.55; Figure 5c), and M0 macrophages were also negatively associated with CD4 memory-activated T cells (r = −0.44) and M1 macrophages (r = −0.41) (Figure 5c).

### 3.5. PCLO Expression in OSCC Tissues

**Figure 6:**
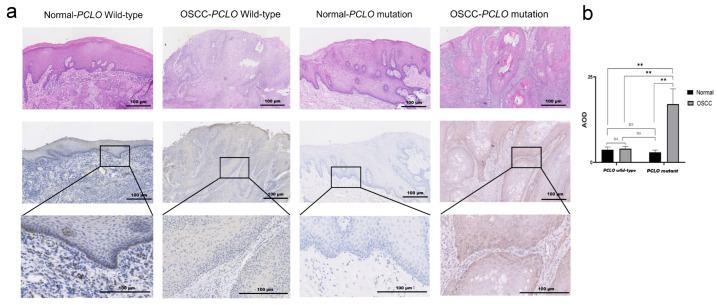
*PCLO* expression in tissues. (**a**) Representative H&E images and corresponding immunohistochemistry images of piccolo expression in adjacent normal epithelium tissues and OSCC tissues from patients with or without *PCLO* mutation. Scale bar, 100 μm. A total of 5 samples were analyzed in each group, with the normal group being normal tissues from patients in the OSCC group. (**b**) AOD of piccolo expression in the normal epithelium and OSCC tissues with or without *PCLO* mutation. Normal group vs. OSCC group, *PCLO* mutation vs. *PCLO* wild-type (two-way mixed ANOVA followed by Bonferroni post hoc test). ** *p* < 0.01; ns, not significant; AOD, average optical density; OSCC, oral squamous cell carcinoma.

Using OSCC tissues from the Peking University School and Hospital of Stomatology (Beijing, China), *PCLO* expression was detected using IHC, and the corresponding H&E images are shown in Figure 6a. *PCLO* expression was considerably higher in OSCC tissues with *PCLO* mutations than in corresponding normal epithelium tissues and OSCC tissues without *PCLO* mutations (*p* < 0.01; Figure 6b). Additionally, in patients with OSCC with *PCLO* mutations, the piccolo protein was mainly distributed in the basal layer of the epithelium (Figure 6b).

## 4. Discussion

The prognosis of patients with OSCC is still poor, although great strides have been made in screening, diagnosis, and treatment [24,25]. A number of studies have emphasized the efficiency of targeted molecular therapy, which can improve the survival rate of patients with OSCC [25,26]. In recent years, there have been a number of studies on gene mutations in OSCC, such as those in *TP53* [27] and *Notch1* [28]. The occurrence of OSCC often involves multiple gene mutations [29]. The present study explored potential biomarkers for clinical prognosis by analyzing mutated genes in OSCC.

The present study included 372 patients with OSCC from TCGA and 171 Indian patients with OSCC from the ICGC database, and 17 common frequently mutated genes were identified in the two databases. The present study further investigated the relationship between these 17 mutated genes and the TMB. Based on the mutation status of the 17 overlapping genes, TCGA samples were divided into a wild-type group and a mutated group to further investigate whether these 17 mutated genes were associated with the TMB. The mutation of 13 genes was associated with the TMB. Survival analysis was subsequently performed for these 13 genes. The *PCLO* mutation group exhibited a worse OS than the wild-type group, while the TMB was significantly greater in patients with mutant *PCLO*, indicating that *PCLO* mutation might be an effective factor for determining the TMB. There are few studies related to *PCLO* in OSCC. Therefore, we continued to explore *PCLO*. GSEA was subsequently performed on *PCLO* to investigate whether *PCLO* might influence immune-related signaling pathways and functions. It revealed that ‘oxidative phosphorylation was activated in the *PCLO* mutant group, which can have an impact on immune cell function. Immune cell infiltration analysis was performed to investigate whether the *PCLO* mutation affected immunity, as the mutation caused changes in the TMB, which in turn is associated with immunity [14,15]. The number of M0 macrophages was reduced in *PCLO*-mutant patients compared with in patients in the *PCLO* wild-type group. These findings were consistent with a previous study, showing that these immune cells and immune-related pathways serve vital roles in the immune response and tumor microenvironment [30].

*PCLO* encodes the piccolo protein, which is a short presynaptic cell-matrix protein [10]. It has been reported that piccolo may be involved in regulating membrane transport, exocytosis and endocytosis [31,32,33]. The piccolo protein regulates the release of neurotransmitters and participates in the formation of the presynaptic cytoskeleton matrix [34,35]. Piccolo protein can be found in brain, pancreatic islet, epithelial and cancer cells [36]. *PCLO* mutations have been found in a number of human tumors, including gastric cancer [37], liver cancer [38], myeloid leukemia [39], diffuse large B-cell lymphoma [34] and colon cancer [40]. It has been reported that silencing *PCLO* can promote the invasion of liver cancer cell lines [41], and *PCLO* tends to be mutated in poorly differentiated liver cancer [38]. It has been reported that knockdown of *PCLO* in esophageal cancer cell lines could inhibit the subcutaneous reproductive capacity of cancer cells in nude mice [10]. Researchers have also injected *PCLO*-knockdown cells into nude mice, and the lung metastasis rate of cancer cells was substantially lower in *PCLO*-knockdown mice than in control mice [10]. The study also revealed that piccolo protein expression was upregulated in *PCLO*-mutant ESCC samples [10]. Two immune subtypes have been reported in HPV^+^ cervical cancer: Immune-H and immune-L. Compared with the immune-L type, the immune-H type exhibits greater immunity, more stromal content, lower tumor purity, proliferative potential, intra-tumour heterogeneity and stemness, higher tumor mutational load, more neoantigens, lower levels of copy number alterations, lower DNA repair activity, and improved overall survival and prognosis. The mutation rate of *PCLO* in the immune-L group was greater than that in the immune-H group [35]. Therefore, we hypothesized that these mutations may change the structural stability of *PCLO*, thus affecting the expression of piccolo. The present study revealed that *PCLO* might be an immune-related gene since the TMB of the *PCLO* mutation group was greater than that of the wild-type group. More antigens are exposed under conditions of high TMB, possibly allowing patients in the *PCLO* mutation group more opportunities to benefit from immunotherapy [42,]. Additionally, the present study showed that piccolo protein expression was upregulated in OSCC with *PCLO* mutations, which is consistent with the results of ESCC research by Zhang et al. [10]. Based on the IHC results, the piccolo protein was centrally expressed in the stratum basale of the *PCLO*-mutated OSCC epithelium, which is also called the stratum germinativum and functions as a stem cell with great proliferative capacity [43]. It has been reported that the piccolo protein promotes cell proliferation and other related effects by regulating *EGFR* signaling [10]. Thus, we hypothesized that there is a similar effect in the stratum basale of the OSCC epithelium, which may shed some light on the poor survival of the *PCLO*-mutated group. Therefore, the present study provides a novel guide for the pathogenesis and therapeutic targeting of patients with OSCC with *PCLO* mutations, which may improve the survival rate and prognosis. Meanwhile, based on the upregulation of PCLO protein expression levels in ESCC, perhaps PCLO protein expression levels also have the potential to serve as an OSCC prognostic indicator.

In addition, M0 macrophage levels were significantly lower in the mutant group than in the wild-type group. A previous study has shown that macrophages are crucial in the immune system and that differentiated macrophages are key factors in the immune response [44]. The reason for the decrease in M0 macrophages may be that apoptotic oral cancer cells activate M0 macrophages to differentiate into M2 macrophages, resulting in low infiltration of M0 macrophages in tumor tissue [45,46,47]. Furthermore, according to the immune cell correlation diagram, a decrease in M0 macrophages increased the number of CD8 T cells and CD4 memory-activated cells. Although no significant differences in CD8 T cells and CD4 memory-activated cells were observed, the negative correlation of M0 macrophages with CD4 memory-activated cells and CD8 T cells may provide some clues for the study of the influence of *PCLO* on OSCC in the future. In the *PCLO* mutation group, several cancer-related pathways, such as the ‘proteasome’ and ‘oxidative phosphorylation’ pathways, were enriched. The former pathway is related to cell cycle regulation, cell differentiation, signal transduction pathways, and immunity, while the latter pathway affects metabolism in multiple cancer types [48]. Some tumor cells exhibit an enhanced dependence on oxidative phosphorylation [49].

The main limitation of the present study was that the ICGC database lacks corresponding clinical data for patients with OSCC, and thus, the associations of prognosis and immune infiltration with *PCLO* mutations were explored using clinical data from only TCGA. It was not possible to verify whether *PCLO* mutations are related to prognosis in Indian patients with OSCC or whether they can cause the same immune response in this population. Although *PCLO* is a frequently mutated gene in Indian patients with OSCC, its effect may be heterogeneous among different ethnic groups. IHC was conducted to validate the relationship between *PCLO* mutation and piccolo protein expression, but the sample size was not large enough. Therefore, the relationship between the expression of genes related to *PCLO* mutation, including genes related to immune infiltration and signaling pathways, and patient prognosis needs to be explored in future experiments. We will also conduct multicenter studies and use in vivo models to overcome these limitations.

## Data Availability

The data generated in the present study are not publicly available due to the protection of patient privacy.

## Supplementary Materials

The following supporting information can be downloaded at: https://www.mdpi.com/article/10.3390/cimb47060426/s1.
