# Supplementary material for: PCLO Is Associated with Tumor Mutational Burden and Immunity in Patients with Oral Squamous Cell Carcinoma
Source: Curr Issues Mol Biol. 2025 Jun 6;47(6):426. doi: 10.3390/cimb47060426 (PMC12192405; doi:10.3390/cimb47060426)
Supplement: Supplementary file 1 [file cimb-47-00426-s001.zip › cimb-3577916-supplementary.pdf]

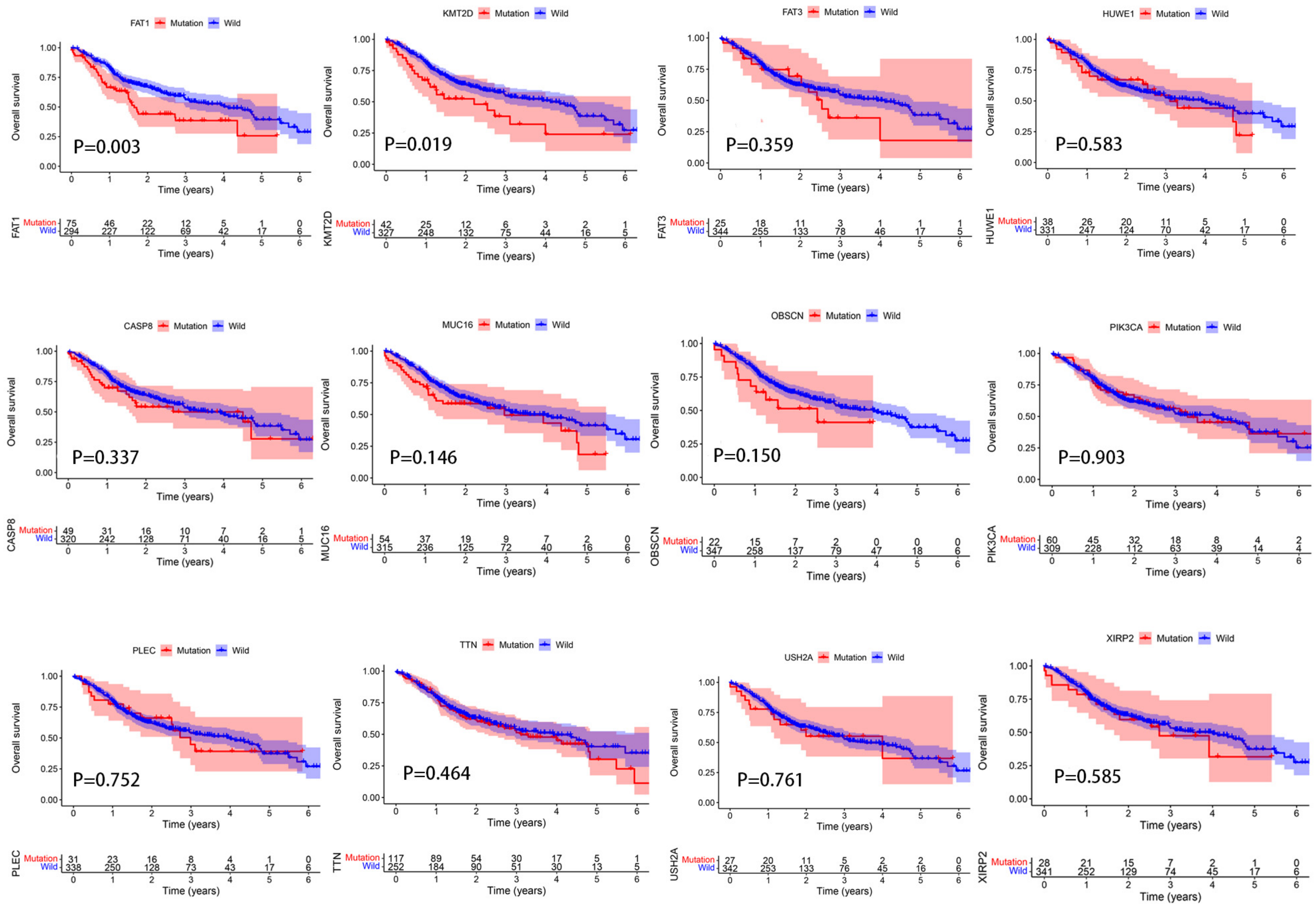

Figure S1. Kaplan-Meier analysis of overall survival based on 12 mutated genes. The P-value is indicated in each plot
